# Peripheral blood (PB) concentrations of neutrophil chemoattractant IL-8 and Th1-related cytokines are elevated during febrile episodes in children with periodic fever, aphthous stomatitis, pharyngitis and adenitis (PFAPA) syndrome

**DOI:** 10.1186/1546-0096-10-S1-A113

**Published:** 2012-07-13

**Authors:** Liora Harel, Eduard Ling, Tirza Klein, Moshe Israeli, Jacob Amir

**Affiliations:** 1 Rabin Medical Center, Petach Tikvah, Israel; 2 Rabin Medical Center, Campus Beilinson, Petach Tikvah, Israel; 3 Schneider Children's Medical Center of Israel, Petach Tikvah, Israel

## Purpose

PFAPA is an autoinflammatory syndrome of childhood characterized by recurrent attacks of fever, aphthous stomatitis, pharyngitis and adenitis. The underlying immune disorder is poorly understood, albeit activation of Th1 related cytokines has been observed. We aimed to compare concentration of proinflammatory cytokines and selected chemokines in PB during and between febrile episodes in children with PFAPA.

## Methods

Twenty three children with PFAPA (8 girls and 15 boys, age 7+1.7 ye, range 5-9 ye), observed at Pediatric Rheumatology Unit of Schneider Children’s Medical Center of Israel, participated in the study. PB was drawn during febrile episodes and between attacks (12 and 18 blood samples, respectively). PB samples of two of febrile children were matched with blood samples of the same children between febrile attacks. Concentration of GM-CSF, INFγ, IL-1β, IL-2, IL-4, IL-5, IL-6, IL-8, IL-10 and TNFα were simultaneously measured in PB using the multiplex ELISA technique.

## Results

PB concentration of IL-8 increased 50-fold (3508.47+2907.16 pg/ml vs 74.36+116.73 pg/ml, respectively, p<0.001) during febrile episodes. Concentrations of IFNγ, IL-1β, IL-2, IL-6 and TNFα were significantly increased during febrile episodes (11.65+6.62 pg/ml, 57.09+1637.91 pg/ml, 10+0.88 pg/ml, 150.79+2243.16 pg/ml and 45.53+119.39 pg/ml vs. 7+12.69 pg/ml, 9+16/8 pg/ml, 10+0.45 pg/ml, 9.47+42.34 pg/ml and 16+8.7 pg/ml, respectively, p<0.001). Concentration of IL10 decreased during febrile episodes (6+1.19 pg/ml vs. 6+2.22, p<0.003), whereas expression of GM-CSF, IL-4, IL-5 remained unchanged.

## Conclusion

PFAPA febrile episodes are characterized by activation of chemokine IL-8, indicating the potential involvement of neutrophils in pathogenesis of the syndrome. Elevation of concentration of proinflammatory cytokines IFNγ, TNFα, IL1β and IL-6 coupled with lack of activation of IL-4 and IL-5 indicate innate immune response with Th1 skewing without involvement of Th2. Expression of eosinophil growth factor IL-5 did not change, indicating the lack of eosinophils’ role in pathogenesis of PFAPA. Decreased expression of suppressor cytokine IL-10 during febrile episode indicates disordered immune regulation.

## Disclosure

Liora Harel: None; Eduard Ling: None; Tirza Klein: None; Moshe Israeli: None; Jacob Amir: None.

